# Using JAK inhibitor to treat cytokine release syndrome developed after chimeric antigen receptor T cell therapy for patients with refractory acute lymphoblastic leukemia: A case report

**DOI:** 10.1097/MD.0000000000025786

**Published:** 2021-05-14

**Authors:** Fu Ming Zi, Long Long Ye, Ji Fu Zheng, Jing Cheng, Qing Ming Wang

**Affiliations:** Department of Hematology, The Second Affiliated Hospital of Nanchang University, Nanchang, Jiangxi, People's Republic of China.

**Keywords:** CAR-T, cytokine release syndrome, Janus Associated Kinases inhibitor

## Abstract

**Rationale:**

Significant concerns about the adverse effects following chimeric antigen receptor T cell (CAR-T) therapy are still remained including cytokine release syndrome (CRS). In rare circumstances, CRS may be refractory to tocilizumab and/or corticosteroids, a new treatment is needed for the management of CRS.

**Patient concerns:**

We present a case of a 20-year-old male patient with acute lymphoblastic leukemia developed CRS after CD19/CD22 bispecific CAR-T treatment.

**Diagnosis:**

The patient was diagnosed with BCR-ABL(P210) positive B-ALL and developed CRS after CD19/CD22 bispecific CAR-T treatment.

**Interventions:**

Tocilizumab and methylprednisolone were administered, unfortunately the patient's symptoms of CRS were still not resolved. Another methylprednisolone and ruxolitinib were administered.

**Outcomes:**

The persistent fever and hypotension of this patient achieved a rapid clinical remission within hours after ruxolitinib administration.

**Lessons:**

Ruxolitinib can be used as an alternative therapeutic approach for severe and refractory CRS without impairing CAR-T amplification and anti-tumor effect.

## 1 Introduction

The treatment of acute lymphoblastic leukemia (ALL) still remains challenging, especially for relapsed and refractory adult ALL, which has a dismal prognosis and has a remission rate of only 29% after standard regimens.^[1]^ The chimeric antigen receptor T cells (CAR-T) therapy, which uses genetically modified T cells to target cancer cells, is an emerging new treatment for hematological malignancies in recent years.^[2–4]^ An anti-CD19 CAR-T therapy, Tisagenlecleucel, had been approved by Food and Drug Administration (FDA)^[5]^ and European Medicines Agency (EMA)^[6]^ for the treatment of children and young adults with refractory or relapsed B cell ALL, and produced high remission rates and durable remissions without additional therapy for those patients. Since the risks associated with CAR-T are substantial, which can lead to ICU-level care and death in some cases, significant concerns are remained about the adverse effects following CAR-T therapy, including cytokine release syndrome (CRS) and CAR-related encephalopathy syndrome (CRES), also known as immune effector cell associated neurotoxicity syndrome (ICANS).^[7,8]^ CRS is a supraphysiologic response that results in the activation or engagement of endogenous or infused T cells and/or other immune effector cells, which can be mitigated in most patients by cytokine blockade such as Tocilizumab (IL-6 receptor antagonist) and corticosteroids.^[9]^ However, it is not sufficiently effective for a minority of patients, therefore, a new treatment for curbing potentially lethal severity CRS is urgently needed in the clinical setting. Ruxolitinib (JAK 1/2 inhibitor), the first FDA approved anti-inflammatory treatment for myelofibrosis and polycythemia vera, has showed a significant effect of inflammatory cytokines reduction in clinical studies.^[10]^ Recent studies have found that it can suppress allergic and immune responses due to its broad anti-inflammatory activity, and can be used for hemophagocytic lymphohistiocytosis (HLH) and glucocorticoid-resistant acute graft-versus-host disease (GVHD) treatment.^[11–14]^ Ruxolitinib is also a potential candidate for severe coronavirus disease 2019 (COVID-19) which is characterized by an exuberant cytokine storm.^[15]^ In a xenograft model, ruxolitinib can prevent CRS after CAR-T therapy without impairing the anti-tumor effect.^[16]^

Therefore, there is a compelling rationale for investigating ruxolitinib as a treatment of CRS. We hereby report a case of severe CRS treated with ruxolitinib after tocilizumab and steroids. In this case, the symptoms of CRS responded quickly, and the patient achieved complete remission. We present the following article in accordance with the CARE reporting checklist.

## 2 Case presentation

**Figure 1:**
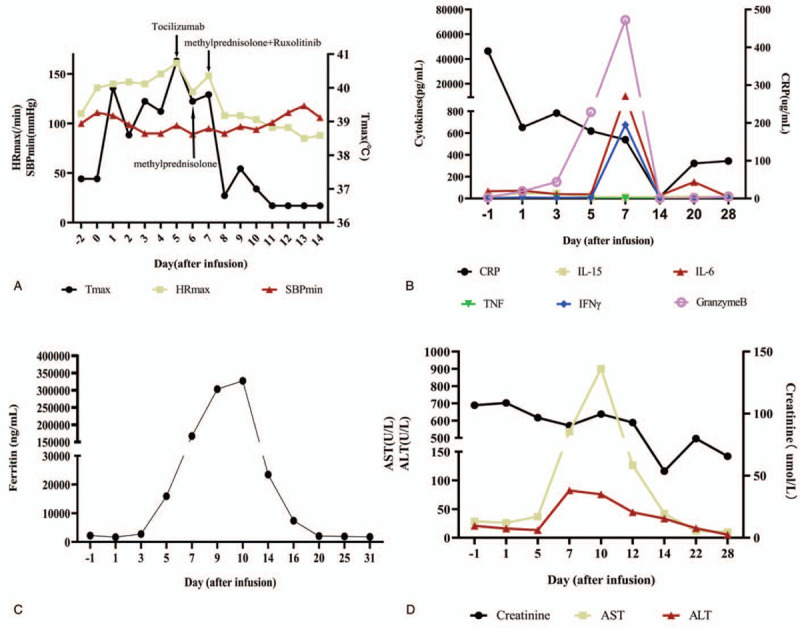
Clinical parameters of the patient. A: The graph shows the patient's maximum temperature (*T*_max_), maximum heart rate (HR_max_), minimum systolic blood pressure (SBP_min_), and the administration of different drugs. B: Serum cytokines levels before and during treatment. C: Ferritin levels before and during treatment. D: The AST/ALT and creatinine. ALT = alanine aminotransferase, AST = aspartate aminotransferase.

**Figure 2:**
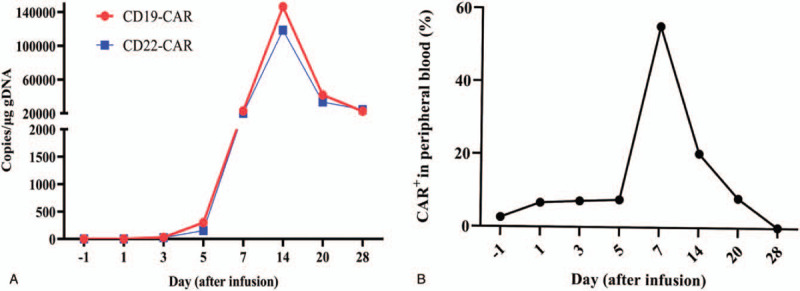
Expansion and persistence of CAR-T cells in vivo. A: copies of CD19 and CD22 CAR in peripheral blood. B: CAR-T percentage in peripheral blood via flow cytometry. CAR-T = chimeric antigen receptor T cell.

A 20-year-old male patient presented to our hospital with lumbago and hematuria in December, 2019. There was no abnormal in his medical history, family, and psycho-social history. At initial diagnosis, the patient had an obvious increase in white blood cells (359.97 × 10^9^/L), and the immunophenotyping via flow cytometry showed that lymphoblasts accounted for 60% of nucleated cells in bone marrow and exhibited the expression pattern of HLA-DR+CD10+CD13+CD19+CD22+CD25+CD33+CD34+CD38+CD123+cCD79a+TdT+. BCR-ABL(P210) transcript was detected to be positive by QRT-PCR. Therefore, the patient was diagnosed with BCR-ABL(P210) positive B-ALL (Normal B cell type, poor prognosis group). The patient was treated with dasatinib combining chemotherapy (VDP), after 3 courses of chemotherapy, complete response (CR) with minimal residual disease (MRD) positive was achieved with 5.85 × 10^4^ BCR-ABL(P210) copies**/**mL. After another courses of chemotherapy (hyper-CVAD) in April 2020, Thr315Ile mutation were detected, thus ponatinib was used instead of dasatinib. However, remission was not achieved. Therefore, the patient was enrolled in a CD19/CD22 bispecific CAR-T therapy clinical trial (NCT04303520). Before conditioning chemotherapy, the lymphoblasts in bone marrow accounted for 95% and the BCR-ABL/ABL(IS) occupied 72.03%, conditioning chemotherapy was initiated 4 days before CAR-T infusion with fludarabine (30 mg/m^2^ from day -4 to -2) and cyclophosphamide (500 mg/m^2^ on day -4 and -3). Then autologous CD19/CD22 bispecific CAR-T was infused (6 × 10^6^/kg on day 0, provided by Shanghai Hrain Biotechnology Co. Ltd.). Because the patient developed fever (maximum 40 °C) and high heart rate (140/min) without other symptoms on day 1, he was treated empirically with antibiotics, nonsteroidal anti-inflammatory agents (diclofenac sodium), and metoprolol. In the following days, fever persisted >3 days with normal procalitonin, but there were continuous increases in serum ferritin and IL-6. His heart rate increased to 161 beat per minute (bpm), the respiratory rates increased to 26 breaths per minute, and the body temperature reached 40.8 °C on day 5, then tocilizumab (400 mg) were administered on day 5, symptoms associated with CRS were resolved rapidly in response to tocilizumab, the body temperature dropped to about 38 °C, but quickly rose to 39.6 °C in 12 hours. The heart rate dropped to about 110 bpm, but also quickly rose to 130 bpm in 12 hours. Concerning that the patient's disease would progress rapidly, methylprednisolone (40 mg) were administered on day 6, the body temperature fluctuated between 37.4 and 39.8 °C with high heart rate (maximum of 148 bpm) in the next 24 hours, and developed a hypotension with a systolic blood pressure of 89 mm Hg. Ferritin and cytokines in plasma including IL-6, IFN-γ, Granzyme B were elevated dramatically (Fig. 1B and C), high aspartate aminotransferase (AST, 535.67 U/L) and alanine aminotransferase (ALT, 82.3 U/L) also indicated liver function impairment (Fig. 1D). Considering the patient's physical condition continued to deteriorate gradually, we started to administer another methylprednisolone (40 mg) and ruxolitinib at a dose of 10 mg every 12 hours on day 7 for 11 days and a dose of 5 mg every 12 hours for next 11 days. On day 8, although the patient developed prolonged activated partial thromboplastin time (APTT, >180 seconds) and elevated D-Dimer (65.98 mg/L), the symptoms associated with CRS were resolved rapidly (body temperature: 36.8 °C, heart rate: 108 bpm, systolic blood pressure: great than 90 mmHg), the levels of cytokines decreased dramatically in the following days (Fig. 1B). The APTT and AST/ALT reached normal level on day 17 and day 22 respectively. No vasopressors or oxygen were required during this period. The expansion of the CAR-T cells in the peripheral blood was sustained (Fig. 2). The patient achieved CR with MRD negative via flow cytometry, while BCR-ABL/ABL(IS) accounted for 5.30% at day 28 after infusion of CAR-T. Timeline of disease course is shown in Fig. 1.

## 3 Discussion

CD19-directed CAR-T has shown significant therapeutic efficacy for relapsed/refractory B-cell ALL with about 80% CR rates, however, 77% to 85% patients after CAR-T infusion would develop a unique treatment-related CRS with a wide constellation of symptoms with multiorgan involvement.^[2,3]^ Although most cases of CRS are self-limited or controllable, some patients can exhibit severe to life-threatening CRS. As is known that CRS is triggered by the activation of T cells on engagement of CARs with cognate antigens expressed by tumor cells, the activated T cells release cytokines and chemokines (including IL-2, soluble IL-2Rα, IFNγ, IL-6, soluble IL-6R, and GM-CSF), as bystander immune cells, the kinetics and exact timing of CRS induction is variable. Nevertheless, IL-6 is a critical cytokine in the progression of CRS,^[17]^ tocilizumab is the first-choice treatment for CRS, rapid clinical stabilization is frequently seen with the use of tocilizumab, which strongly implicating cytokines, especially IL-6.^[8]^ Some patients may be refractory to tocilizumab, repeated administration of corticosteroids is the reserved treatment for those patients. The corticosteroids may potentially inhibit CAR-T persistence and the therapeutic efficacy, and in rare circumstances, CRS may be refractory to tocilizumab and/or corticosteroids, a new treatment is needed for optimal approaches for the management of CRS other than tocilizumab.

For patients who do not respond to IL-6 blockade, another anti-cytokine drug, Siltuximab (an IL-6 antagonist), is encouraged for the treatment of CRS as it blocks IL-6 directly. It may be used as the second line treatment following tocilizumab or the third line treatment after tocilizumab and corticosteroids, but it should be noted that siltuximab was not sufficiently effective as a treatment for refractory and severe CRS which proved fatal in 2 cases of refractory and severe CRS.^[18,19]^ To date, no detail is available with regards to the line of use of siltuximab. IL-1 is considered to play a role in triggering CRS. Anakinra, which inhibits the activity of IL-1 may theoretically reduce CRS after CAR-T cell therapy,^[20,21]^ this approach has not yet been tested in clinical trials, there are no published data of anakinra for this indication.

IL-6 signaling pathway can ultimately result in the activation of the JAK/STAT pathway which plays a major role in transferring of signals from cell-membrane receptors to the nucleus and is essential for a wide range of cytokines, thus ruxolitinib (JAK1/2 inhibitor) maybe a potential candidate for the treatment of CRS as it can suppress immune responses for its broad anti-inflammatory activities and was used to treat myelofibrosis, polycythemia vera, HLH, and GVHD by significantly reducing inflammatory cytokines.^[10–14]^ In an acute myeloid leukemia (AML) xenograft model, ruxolitinib can prevent the development of severe CRS without impairing the anti-tumor effect of CD123-directed CAR-T therapy,^[16]^ these indicate that ruxolitinib could also be used in clinical practice for the management in severe CRS. In a recent study, ruxolitinib has been shown to potently suppress CAR-T-cell cytotoxicity in vitro, while the mechanisms are unknown.^[22]^ There are several new papers showing effect of ruxolitinib in CRS. In a case report, ruxolitinib was used to treat steroid-refractory CRS without significant impact on the antileukemic effect of CAR-T therapy, which suggest that adjuvant ruxolitinib therapy may be an alternative therapeutic approach for the management of CRS.^[23]^ In another case, Grade 3 CRS occurred and was managed by a ruxolitinib-based CRS management.^[24]^ In this report, our patient suffered from fever (maximum 40 °C) on day 1 and persistent fever lasting greater than 3 days with a continuous increase in cytokines. Tocilizumab were administered on day 5, symptoms associated with CRS were resolved rapidly in response to tocilizumab, the body temperature dropped to about 38 °C, but quickly rose to 39.6 °C in 12 hours. On day 6 methylprednisolone (40 mg) were administered, the body temperature fluctuated between 37.4 and 39.8 °C in the next 24 hours, and developed a hypotension with a systolic blood pressure of 89 mm Hg (Fig. 1A), we administered another methylprednisolone (40 mg) and ruxolitinib at a dose of 10 mg every 12 hours, although we cannot completely exclude the potential effect of methylprednisolone, the persistent fever and hypotension of this patient achieved a rapid clinical remission within hours after ruxolitinib administration, which is consistent with the above case report. Furthermore, this patient achieved CR on day 14 after infusion of CAR-T. Overall, our findings from this patient and the case reports from other groups indicate that ruxolitinib may be an important alternative therapeutic approach for the management of CRS, larger patient cohorts clinical trials would be needed to further verify this result.

## Author contributions

**Analysis and interpretation of data:** Long Long Ye, Ji Fu Zheng.

**Data curation:** Ji Fu Zheng.

**Investigation:** Long Long Ye, Jing Cheng.

**Patient data collection/Data acquisition:** Jing Cheng.

**Supervision:** Qing Ming Wang.

**Writing – original draft:** FuMing Zi.

**Writing – review & editing:** Qing Ming Wang.

**Writing and/or critical revision of the manuscript:** Fu Ming Zi, Qing Ming Wang.
